# Liver Stiffness and Steatosis Measurements with iLivTouch and FibroScan: A Comparative Study

**DOI:** 10.5152/tjg.2024.23531

**Published:** 2024-08-01

**Authors:** Mübin Özercan, Zeynep Melekoğlu Ellik, Ayhan Parmaksız, Mesut Gümüşsoy, Serkan Duman, Necati Örmeci

**Affiliations:** 1 Department of Gastroenterology, Fırat University School of Medicine, Elazığ, Türkiye; 2 Department of Gastroenterology, Karaman State Hospital, Karaman, Türkiye; 3 Department of Biostatistics, İstanbul Health and Technology University, İstanbul, Türkiye; 4 Department of Gastroenterology, Gaziantep Ersin Arslan State Hospital, Gaziantep, Türkiye; 5 Department of Gastroenterology, Toros State Hospital, Mersin, Türkiye; 6 Department of Gastroenterology, İstanbul Health and Technology University, İstanbul, Türkiye

**Keywords:** Elastography, iLivTouch, FibroScan, stiffness, steatosis

## Abstract

**Background/Aims:**

The presence of liver fibrosis is the most important indicator of progression to cirrhosis. Noninvasive measurement of liver stiffness is crucial for detecting fibrosis. Vibration-controlled transient elastography is one of the most useful methods for this purpose. We aimed to compare the liver stiffness and steatosis measurements with iLivTouch© and the FibroScan© elastography devices

**Materials and Methods:**

Two hundred thirty-seven consecutive adult patients with chronic hepatitis were included in the study. The liver stiffness and steatosis were measured with iLivTouch and FibroScan on the same day. Thirty-one patients had liver biopsies on the same day with elastography procedures. The diagnostic performances of iLivTouch and FibroScan were compared to aspartate aminotransferase to platelet ratio index (APRI), Fibrosis-4 (FIB-4), and nonalcoholic fatty liver disease fibrosis score (NFS).

**Results:**

The liver stiffness measurements obtained using iLivTouch and FibroScan had median value of 10.3 (ranging from 2.9 to 46.3) and 7.2 (ranging from 2.5 to 75), respectively. The mean steatosis measurements using ultrasound attenua­tion parameter with iLivTouch were 245.51 ± 45.79, while the mean controlled attenuation parameter measurements using FibroScan were 259.37 ± 75.0. In subgroup analysis, the AUC of iLivTouch on detecting signiicant fibrosis [0.83, (*P* = .002)] was minimally higher than other noninvasive methods [0.82 for NFS (*P* = .003), 0.80 for FibroScan (*P*= .006), 0.68 for FIB-4 (*P* = .089), and 0.53 for APRI (*P* = .76)].

**Conclusion:**

The stiffness and steatosis measurements with iLivTouch and FibroScan were not similar. The accuracy of iLivTouch in detecting significant and advanced fibrosis was minimally higher. Large clinical trials are necessary to support these findings.

**Main Points**

- Transient elastography is a recommended approach in worldwide guidelines for assessing liver stiffness as a predictor of fibrosis. The iLivTouch device was found to be comparable to the widely recognized elastography equipment, FibroScan.
- The analysis of the stiffness and attenuation parameters of the iLivtouch and FibroScan devices indicated that the outcomes are not similar.
- Subgroup analysis revealed that the accuracy of iLivTouch is minimally higher than FibroScan and the other non-invasive scores in detecting fibrosis.

## Introduction

One and a half billion people are thought to have chronic hepatitis worldwide, and hepatitis B and hepatitis C are 2 major causes of cirrhosis, followed by alcohol and nonalcoholic steatohepatitis (NASH).^1,2^ Cirrhosis and hepatocellular carcinoma are the most serious and mortal complications of chronic hepatitis.^3^ In 2017, almost 1.32 million people died due to cirrhosis and this was equal to 2.4% of all-cause deaths. The number of people dying from cirrhosis is increasing every year.^2^ Therefore, it is of great importance to detect the presence of cirrhosis in patients with chronic hepatitis.

Globally, the estimated overall prevalence of image-based nonalcoholic fatty liver disease (NAFLD) was 25.24%. Among them, estimated biopsy-proven NASH prevalence was 59.1% and the incidence of fibrosis progression was approximately 41%.^4^ In Türkiye, Cappadocia cohort study revealed a higher prevalence of NAFLD (60.1%).^5^

The gold standard test used to detect the degree of steatosis and fibrosis in the liver is liver biopsy. However, liver biopsy is an invasive method and has high rates of sampling error and interobserver discordance.^6^ Therefore, noninvasive scoring methods and elastography methods have been developed in recent years to assess liver pathologies, especially the presence of fibrosis.^7^

Liver stiffness measurement (LSM) and controlled attenuation parameter (CAP)/ultrasound attenuation parameter (UAP) are measured with vibration-controlled transient elastography (VCTE), for predicting the diagnosis and staging of liver fibrosis and steatosis, respectively.^8^

The FibroScan© (Echosens, Paris, France) is the first transient elastography device. The iLivTouch© FibroTouch (Wuxi Hisky Medical Technology Co., Ltd., Wuxi, China) has been used in clinical applications since 2013 and compared with other noninvasive methods for the assessment of steatosis and fibrosis in the liver. Steatosis is measured as UAP in iLivTouch.

Vibration-controlled transient elastography methods have some limitations. Acute hepatitis, nonfasting, congestion, cholestasis, and inflammation can be causes of false positivity. Additionally, obesity, narrow intercostal space, and ascites affect the reliability of the results. Cutoff values of stiffness were found adjusted according to the etiology of liver disease.^9^ Controlled attenuation parameter/UAP cutoffs vary according to the etiology of steatosis and are more accurate in recognizing steatosis in viral hepatitis.^10^ Grading steatosis according to CAP/UAP score was also found inadequate in NAFLD.^10^

The aim of the study was the comparison of liver stiffness and attenuation parameter measurements of 2 VCTE devices, FibroScan, and iLivTouch.

## Materials and Methods

### Study Design

The study included 237 consecutive patients that VCTE was performed with FibroScan and iLivTouch, in Ankara University Hepatology Outpatient Clinique, a tertiary academic center for liver, between April 2019 and March 2020. The indications for transient elastography were evaluating fibrosis in patients with chronic liver diseases (higher transaminases more than 6 months), suspected NAFLD, and before liver biopsy. Liver stiffness and steatosis were measured with both FibroScan and iLivTouch on the same day. Age, gender, weight, and height parameters were collected from all patients before elastography and the laboratory results [alanine aminotransferase (ALT), aspartate aminotransferase (AST), alkaline phosphatase (ALP), gamma-glutamyl transpeptidase (GGT), albumin, bilirubin, fasting glucose, platelet count, international normalized ratio (INR)] obtained on the same day with elastography measurements and transabdominal ultrasounds or cross sectional imagings were evaluated retrospectively. The exclusion criteria were patients younger than 18 years old, liver mass or any malignancy, ascites, extrahepatic cholestasis, acute hepatitis, and pregnancy. Six participants were removed from the study due to an absence of reliable measurements using FibroScan. Biopsies were obtained with a 16 gauge core-biopsy needle by expert gastroenterology fellows and re-evaluated by a blind hepato-pathologist according to Metavir scores.

FibroScan and iLivTouch were performed by 2 gastroenterology fellows experienced with more than 100 transient elastographic measurements who were blind to the other measurement results. Each patient was laid in a supine position with the right hand placed under the head during the measurement. After smearing the coupling agent, the probe was applied to the skin on the seventh-ninth intercostal spaces. The pressure was maintained within the permitted range of devices while the probe was in the vertical position. The FibroScan M probe was used in most patients; however, the XL probe was used in case of invalid results with the M probe, especially in patients with a higher body mass index (BMI). The validity criteria of elastography were interquartile range (IQR)/Med <30% and consecutive 10 valid measurements of the device.

First, statistical analyses were performed with the entire data set (study 1: n = 231). Then, since LSM values above 15 kPa were considered advanced fibrosis for both devices, these values were excluded and agreement analyses were performed with 185 cases (study 2). Thus, the measurements in the range of fibrosis classification from F0 to F4 were compared.

This study was approved by the Institutional Ethics Review Committee at Ankara University (approval number: 2022/313, date: June 10, 2022). Written informed consent was obtained from each patient.

### Statistical Analysis

Qualitative variables were summarized with count and percentage, and quantitative variables were summarized with mean, standard deviation, median, minimum, and maximum. Paired samples were compared using a paired *t*-test. Evaluation of agreement between 2 devices was conducted using Cohen’s kappa statistics in qualitative variables and using Deming regression, Bland–Altman analysis, and concordance correlation coefficient in quantitative variables.

These methods are frequently used to examine 2-device agreements for quantitative measurements. For Deming regression analysis, an agreement between devices is considered if the 95% confidence interval of the constant coefficient contains 0 and the 95% confidence interval of the slope coefficient contains 1. In the Bland–Altman plot, there are the averages of the measurements obtained with the 2 devices for each patient on the x-axis and there are the differences between the measurements on the y-axis. It is expected that the differences between the 2 device measurements should be randomly distributed (without any specific trend or shape/pattern) around 0 and within the limits of ±1.96 times the standard deviation of the differences (±1.96×SD). The concordance correlation coefficient (CCC) is used to test for separation from a scatter with a slope of 1 and an agreement between devices is considered poor below the 0.90 CCC.

The diagnostic performances of iLivTouch, FibroScan, and other noninvasive stiffness scores such as AST to platelet ratio index (APRI), Fibrosis-4 (FIB-4), and NAFLD fibrosis score (NFS) were determined with receiver operating curve (ROC) analysis in the liver biopsy subgroup. Statistical Package for the Social Sciences (version 26) and MedCalc for Windows (version 20) were used for statistical and graphical analysis. All reported *P*-values are 2-sided, and statistical significance was defined as *P* < .05.

## Results

Two hundred thirty-one patients were included in the study. The descriptive features of patients are shown in Table 1.

Comparisons were made for paired FibroScan and iLivTouch measurements using paired-samples *t*-test. Statistically significant differences were found between iLivTouch and FibroScan measurements for the paired samples regarding LSM, CAP/UAP, and IQR/MED (Table 2). Regarding LSM, iLivTouch measurements were on average 1.63 kPa (95% CI: 0.69-2.57) higher than FibroScan measurements (*P* = .001). FibroScan CAP measurements were on average 13.59 dB/m (95% CI: 5.47-21.71) higher than iLivTouch UAP measurements (*P* = .001). Regarding IQR/MED, FibroScan measurements were on average 2.01 units (95% CI: 0.42-3.6) higher than iLivTouch measurements (*P* = .014). This showed that iLivTouch measurements were more reliable than FibroScan according to IQR/MED ratios.

Cohen’s *κ* was run to determine if there was an agreement between 2-device classifications in invalid measurements of stiffness. There was poor agreement between the 2 devices, *κ* = 0.144 (95% CI, .018-.270), *P* = .001 (Table 3). This indicated that 10 valid measurements were obtained earlier with iLivTouch.

### Study 1

**Figure 1.**
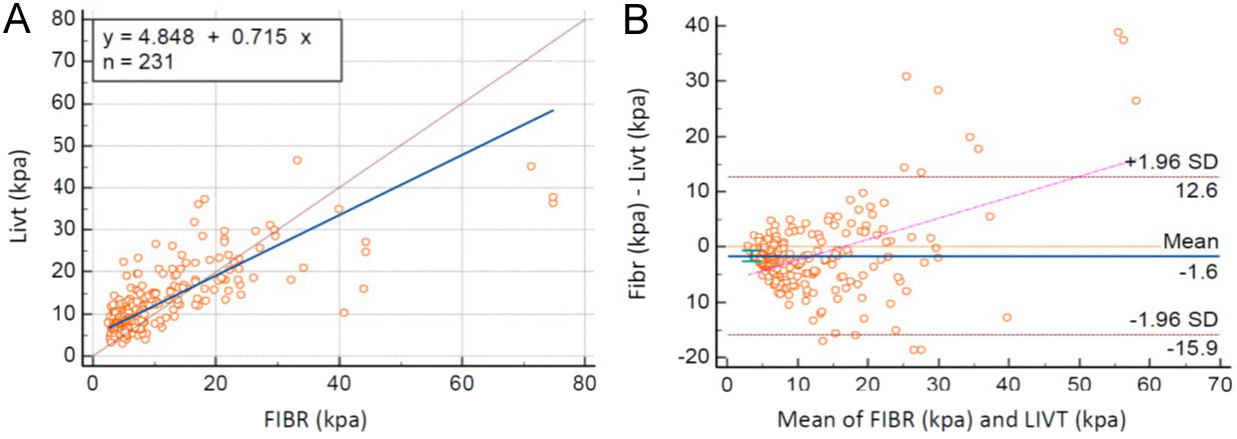
Comparison of stiffness measurements of devices for study 1. (A) Deming regression analysis. (B) Bland–Altman plot.

In study 1 (n = 231), Deming regression analysis revealed that results were not similar according to constant coefficient and slope coefficient (Table 4). Additionally, the scatter plot was evaluated (Figure 1), and it was noticed that there was a considerable proportional bias, and the CCC = 0.70 was lower than 0.90. The non-similarity of stiffness measurements was also provided by these analyses.

The Bland–Altman analysis revealed that the mean of differences was −1.6, and the difference was statistically significant. The Bland–Altman plot showed that at the high measurement values, the differences between the devices increased (a funnel shape was formed), and there was a slope on the graph (Figure 1). Therefore, the difference in measurements with devices was considered significant.

### Study 2 (Liver Stiffness Measurement <15 kPa for Both Devices)

**Figure 2.**
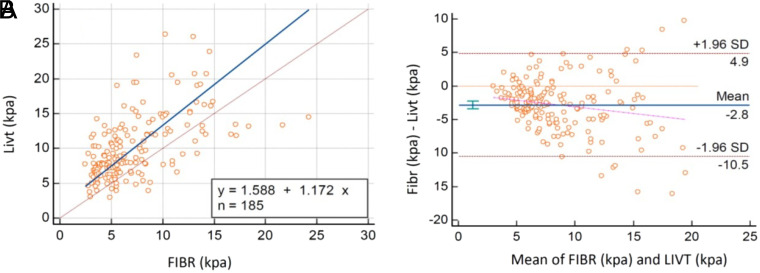
Comparison of stiffness measurements of devices for study 2. (A) Deming regression analysis. (B) Bland–Altman plot.

Deming regression analysis showed that stiffness results were correlated according to the constant coefficient and slope coefficient (Table 4). However, scatter plots showed a regression line nearly parallel to the reference line with a constant difference (Figure 2). As 95% CIs were relatively wide, the calculated CCC = 0.46 was lower than 0.90. Additionally, in the Bland–Altman analysis, the mean of differences was −2.8, and the confidence interval did not include 0 (Figure 2). Therefore, the difference in measurements with devices was considered significant.

Overall, these studies revealed that the stiffness measurements of devices were poorly correlated and the agreement was not within acceptable levels.

### Comparison of Attenuation Parameter Results

Comparison of CAP (FibroScan) and UAP (iLivTouch) scores was evaluated with Deming regression analysis and Bland–Altman analysis. Outlier results (n = 7) were excluded (Table 5).

**Figure 3.**
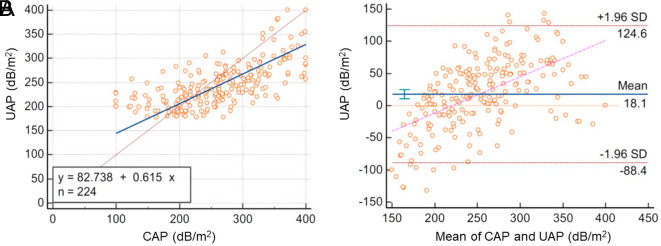
Comparison of steatosis measurements (dB/m^2^) of devices. (A) Deming regression analysis. (B) Bland–Altman plot.

In Deming regression analysis, the attenuation parameter result of devices were not found to be similar (Table 5). Additionally, the scatter plot was evaluated and proportional bias was considered (Figure 3), and the calculated concordance CCC = 0.50 was lower than 0.90. These analyses provided the difference between the results.

In the Bland–Altman analysis, the mean of differences was 18.1 dB/m, and the 95% confidence interval did not include 0. The standard deviation of UAP scores was lower than CAP and additionally, the variance ratio showed that UAP scores had narrower ranges. The Bland–Altman plot showed that the CAP values were lower than the UAP values at the low measurement level, in contrast, the CAP values were higher than the UAP values at the high measurement level (Figure 3). Therefore, the difference in measurements of attenuation parameters was considered significant.

### Accuracy of Noninvasive Fibrosis Tests

Thirty-one participants had liver biopsies soon after FibroScan and iLivTouch measurements. In subgroup analysis, histopathologic assessment of fibrosis was considered the gold standard and compared with LSM (FibroScan and iLivTouch) and APRI, FIB-4, and NFS. The most common etiology of chronic liver disease was NAFLD (n = 13), followed by hepatitis B virus (n = 9).

According to the Metavir scoring method, the distribution of fibrosis levels on histological assessment was as follows: 11 (35.4%) patients with F0, 8 (25.8%) patients with F1, 4 (12.9%) patients with F2, 5 (16.1%) patients with F3, and 3 (9.6%) patients with F4.

The mean LSM values of FibroScan and iLivTouch were 12.45 ± 13.2 and 12.36 ± 6.1, respectively. On detecting significant fibrosis (Metavir ≥2), the diagnostic accuracy of FibroScan (AUROC of 0.80; *P* = .006), NFS (AUROC 0.82; *P* = .003), and iLivTouch (AUROC of 0.83; *P* = .002) measurements were higher than APRI (AUROC of 0.53; *P* = .760) and FIB-4 (AUROC of 0.68; *P* = .089) scores. At a cutoff value of 7.25 kPa, FibroScan demonstrated a sensitivity of 85.7% and specificity of 66.7%. Meanwhile, iLivTouch, at a cutoff value of 7.85 kPa, exhibited a sensitivity of 92.9% and a specificity of 60%.

**Figure 4.**
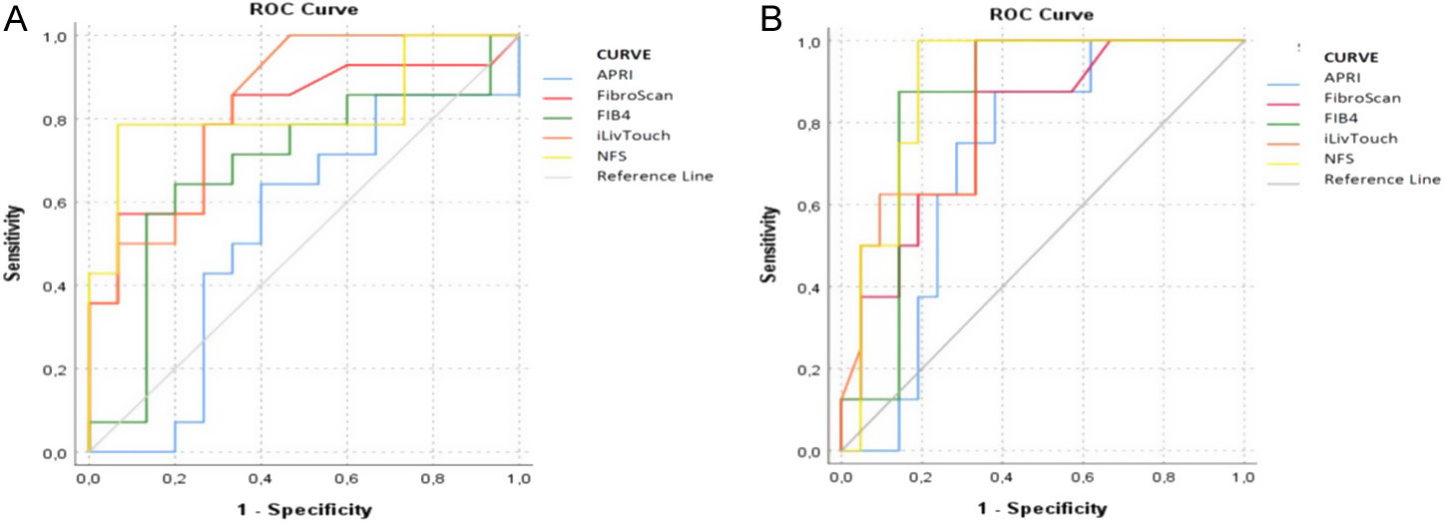
Receiver operating curve analysis of Fibroscan (kPa), iLivtouch (kPa), AST to platelet ratio index, Fibrosis-4, and nonalcoholic fatty liver disease fibrosis score according to significance (A) and advanced (B) fibrosis.

Diagnostic performances of detecting advanced fibrosis (Metavir ≥3) were higher in NFS (AUROC 0.89; *P* = .001), iLivTouch (AUROC of 0.84; *P* = .004), FIB4 (AUROC of 0.82; *P* = .004), and FibroScan (AUROC of 0.78; *P* = .019) than APRI score (AUROC of 0.71; *P* = .079) (Figure 4). The FibroScan cutoff value of 8.5 kPa demonstrated a sensitivity of 87.5% and a specificity of 66.7%. The iLivTouch device achieved a sensitivity of 100% and a specificity of 66.7% when a cutoff value of 11 kPa was used.

## Discussion

This is the first study comparing the measurements of FibroScan and iLivTouch (FibroTouch) devices in Europe. The stiffness and attenuation parameter measurements of the devices were found to be significantly different in the study. The probe of iLivtouch affected the reliability of results according to IQR/Med and low numbers of invalid measurements. In subgroup analysis, stiffness results of iLivTouch tended to be more accurate than FibroScan results.

FibroScan was introduced in 2003 and has become a reference for many hepatologists to stratify chronic liver diseases according to LSM.^11^ Many studies are comparing FibroScan with other elastography methods (ultrasound elastography devices, MR elastography) and variable results were reported.^12,13^ iLivTouch is a newer device based on similar technical aspects of FibroScan and provides noninvasive liver stiffness and steatosis measurements as kPa and UAP, respectively. Studies showed that devices yield comparable findings and show no significant difference in LSM. In our study, we found different results with FibroScan and iLivTouch for both stiffness and attenuation parameters. iLivTouch measurements were on average 1.63 kPa higher than FibroScan measurements. Additionally, CAP measurements with FibroScan were on average 13.59 dB/m higher than UAP measurements with iLivTouch. The differences were statistically significant. The other studies comparing the FibroScan and iLivTouch were analyzed with *t*-test, correlation analysis, and Bland–Altman.^14,15^ More than these tests are required to compare devices. Therefore, we have analyzed data with paired *t*-tests, Bland–Altman, concordance correlation coefficient, and also Deming regression analysis, which are used for comparative studies. In line with our research, Ng et al^16^ reported a correlation coefficient of 0.70 between iLivTouch and FibroScan. However, the overall LSM measurements of the devices were significantly different in the study.

Interquartile range/MED ratios of FibroScan measurements were on average 2.01 units higher than iLivTouch measurements (*P* = .014). Therefore, the validity and reliability rate of iLivTouch measurements were higher than measurements with FibroScan. The dynamic probe of iLivTouch might cause this difference. The probe adjusts positioning and depth of measurement according to the distance between the skin and the liver capsule.

FibroScan was unable to measure 6 participants, even with an XL probe, whereas iLivTouch successfully measured all participants in our study. These participants were obese (BMI = 31.05 ± 10.9). Therefore, the adjustable probe of iLivTouch seems more effective than FibroScan probes, especially in obese patients. Body masss index and waist circumference affected the number of valid measurements during the FibroScan. Patients who lacked 10 valid measurements were reported to be obese (BMI > 35.6), and their waist circumference was also higher (114 ± 14 cm).^17^ The number of invalid measurements during the completion of 10 consecutive valid measurements was lower for iLivTouch (*P* = .001).

Body weight and height are necessary for beginning measurement with iLivTouch, and previous studies showed a significant association between UAP and BMI. Some formulations were also theorized on this relation as UAP = 3.02 × BMI + 186 or UAP = 3.78 × BMI + 146.^15^ In our study, the correlation between UAP and BMI (rho = 0.716, *P* < .001) was higher than CAP with BMI (rho = 0.466, *P* < .001). The formulation results (271.78 ± 17.82) were significantly different from the values of iLivTouch UAP (247.81 ± 47.17) (*P* < .001).

Liver stiffness measurement cutoff values of fibrosis vary in different causes. In NAFLD, cutoff values were reported as 5.8-9.0 kPa, 7.9-9.7 kPa, and 10.3-13.6 kPa for F2, F3, and F4 fibrosis, respectively.^8,11^ According to the recommended cutoff values, when the cutoff value is over 7.9 kPa, liver biopsy is suggested in patients with NAFLD.^6,10,17, 18^ In hepatitis B, cutoff values were reported as 5.85-8.8 kPa for F2, 7-13.5 kPa for F3, and 9-16.9 kPa for F4.^19^ Gatos et al^20^ reported that the AUROC of VCTE was 0.96 for both F3 and F4 in patients with chronic liver diseases. In chronic liver diseases, AUROC of transient elastography was found in the range of 0.79-0.87 for >F2; 0.76-0.98 for >F3, and 0.91-0.99 for F4.^21^ Our study included consecutive patients regardless of etiology; therefore, this was considered one of the major limitations of the study.

Diagnostic accuracy (AUROC) of steatosis in FibroScan was found to be 0.76 in patients with NAFLD.^22^ Cutoff values were reported in the range of 232.5-294 dB/m for steatosis 1 (S1); 255-310 dB/m for S2, and 280-331 dB/m for S3. Most studies reported higher than 0.80 area under he curve (AUC) for each steatosis level.^23^ In our study, only 10 patients had steatosis in liver biopsy; therefore, the accuracy of attenuation parameters could not be analyzed.

The stiffness measurements of FibroScan and iLivTouch were compared with noninvasive fibrosis scores (FIB-4 and APRI) and ARFI. Results of FibroScan and iLivTouch were similar, and their accuracy in detecting fibrosis was higher than the other noninvasive methods.^24,25^ Their results were significantly correlated with histologically classified fibrosis.^25-28^ It was reported that cutoff values were affected by the etiology of liver disease.^26,29^ Area under the curve scores with iLivTouch were reported as 0.84 for >F1; 0.85 for >F2; 0.90 for >F3, and 0.87 for F4.^29^ In our study, the results of the subgroup analysis demonstrated that the sensitivity of iLivTouch for detecting significant fibrosis was found to be 92.9%, which was higher than that of FibroScan at 85.7%. Moreover, the sensitivity of iLivTouch was observed to be 100% in identifying severe fibrosis. The AUC for >F2 was 0.83 in iLivTouch and 0.80 in FibroScan; for >F3 AUC was 0.84 in iLivTouch and 0.78 in FibroScan.

The small number of patients with liver biopsy is an important limiting factor in the interpretation and generalization of the data obtained. Another main limitation of the study was the inclusion of participants with variable etiology in liver pathology because the cutoff values of stiffness measurements could be affected by etiology. Additionally, we had no data about the other chronic systemic diseases of participants.

Elastography is an accepted method for evaluating liver fibrosis. The measurements with FibroScan and FibroTouch/iLivTouch were not found to be similar. Prospective studies with a high number of patients and analyzed with more appropriate statistical tests are necessary to show the similarity or differences between devices. It might be necessary to define the cutoff values for each device separately.

## Figures and Tables

**Table 1.** Patient Characteristics

n	231
Age (minimum–maximum)	51.55 ± 14.15 (18-84)
Gender, male (%)/female (%)	112 (48.5%) / 119 (51.5%)
BMI, kg/m^2^, mean ± SD	28.17 ± 9.78
Etiology of liver disease	
NAFLD/NASH, n (%)	62 (26.8)
Hepatitis B, n (%)	53 (22.9)
Cryptogenic, n (%)	13 (5.6)
Primary biliary cholangitis, n (%)	9 (3.9)
Autoimmune hepatitis, n (%)	7 (3)
Hepatitis C, n (%)	3 (1.3)
Alcoholic hepatitis, n (%)	2 (0.9)
Others, n (%)	82 (35.1)
APRI, med (minimum–maximum)	0.39 (0.04-6.29)
FIB-4, med (minimum–maximum)	1.28 (0.1-12.1)
NFS, med (minimum–maximum)	−1.77 (-8 - 3.77)
FibroScan	
LSM, med (minimum–maximum)	7.2 (2.5-75)
LSM, mean ± SD	11.28 ± 11.05
CAP, mean ± SD	259.37 ± 75.0
IQR, med (minimum–maximum)	1.2 (0.2-33)
IQR/Med, med (minimum–maximum)	16 (2-77)
LivTouch	
LSM, med (minimum–maximum)	10.3 (2.9-46.3)
LSM, mean ± SD	12.91 ± 7.9
UAP, mean ± SD	245.51 ± 45.79
IQR, med (min-max)	1.6 (0.1-64)
IQR/Med, med (min-max)	14 (2-43)

BMI, body mass index; CAP, controlled attenuation parameter; IQR, interquartile range; LSM, liver stiffness measurement; Med, median; NAFLD, nonalcoholic fatty liver disease; UAP, ultrasound attenuation parameter.

**Table 2.** Comparison of Paired Measurements of FibroScan and iLivTouch Devices

	n	FibroScan	iLivTouch	Mean of Differences	95% CI for MoD	*t*	*P*	*r*
LSM	231	11.28 ± 11.06	12.91 ± 7.9	−1.63 ± 7.27	−2.57 to −0.69	−3.408	.001	0.754
CAP/UAP	229	258.92 ± 75.27	245.33 ± 45.81	13.59 ± 62.37	5.47-21.71	3.297	.001	0.562
IQR/MED	226	17.76 ± 9.15	15.76 ± 9.78	2.01 ± 12.13	0.42-3.6	2.485	.014	0.180

CAP, controlled attenuation parameter; IQR, interquartile range; LSM, liver stiffness measurement; MoD, mean of differences; r, Pearson correlation; UAP, ultrasound attenuation parameter.

**Table 3.** Agreement Between FibroScan Invalid and iLivTouch Invalid Cases

		iLivTouch Invalid			Total	Kappa	*P*
		0-5 Invalid	6-10 Invalid	11 Invalid			
FibroScan invalid	0-5 invalid	137 (65.2)	10 (4.8)	0 (0)	147 (70)	0.144	.001
	6-10 invalid	24 (11.4)	3 (1.4)	0 (0)	27 (12.9)		
	11 invalid	26 (12.4)	5 (2.4)	5 (2.4)	36 (17.1)		
	Total	187 (89)	18 (8.6)	5 (2.4)	210 (100)		

**Table 4.** Agreement Analysis in Stiffness Measurements by iLivTouch and FibroScan

Parameter	Study 1		Study 2	
	Intercept	Slope	Intercept	Slope
Coefficient	4.84	0.71	1.58	1.17
Std. error	0.93	0.10	1.29	0.20
95% CI for coefficient	2.99 to 6.69	0.51 to 0.91	−0.96 to 4.14	0.76 to 1.57
Regression equation^†^	y = 4.84 + 0.71x		y = 1.58 + 1.17x	
Correlation coefficient, r	0.75		0.57	
95% CI for r	0.69 to 0.80		0.46 to 0.66	
CCC	0.70		0.46	
95% CI for CCC	0.64 to 0.75		0.36 to 0.54	
Descriptive Statistics of Stiffness Measurements of iLivTouch and FibroScan				
**Variable**	FIBR	LIVT	FIBR	LIVT
Sample size (n)	231		185	
Lowest value (kPa)	2.5	2.9	2.5	2.9
Highest value (kPa)	75	46.3	24.2	26.4
Arithmetic mean (kPa)	11.28	12.91	7.21	10.04
95% CI for the arithmetic mean	9.84 to 12.71	11.88 to 13.93	6.65 to 7.77	9.38 to 10.69
Median (kPa)	7.2	10.3	5.9	8.6
95% CI for the median	6.11 to 8.00	9.11 to 11.98	5.50 to 6.43	7.90 to 9.56
Standard deviation	11.05	7.90	3.86	4.53
Relative standard deviation	0.98 (98.02%)	0.61 (61.21%)	0.53 (53.63%)	0.45 (45.15%)
Variance ratio	1.95		0.72	

CCC, concordance correlation coefficient; FIBR, FibroScan; LIVT, iLivTouch.

†Deming rRegression, x: FIBR(kPa) and y: LIVT(kPa)

**Table 5.** Agreement Analysis in Controlled Attenuation Parameter (FibroScan) and Ultrasound Attenuation Parameter (iLivTouch) Scores on Steatosis

Parameter	Intercept	Slope
Coefficient	82.73	0.61
SE	14.78	0.056
95% CI for coefficient	53.61-111.86	0.50-0.72
Regression equation^†^	*y* = 82.73 + 0.61*x*	
Correlation coefficient, *r*	0.66	
95% CI for *r*	0.58-0.73	
Concordance correlation coefficient	0.56	
95% CI for CCC	0.49-0.63	
**Descriptive Statistics of CAP and UAP**		
**Variable**	**CAP**	**UAP**
Sample size	224	
Lowest value	100	177
Highest value	400	400
Arithmetic mean	262.24	244.13
95% CI for the arithmetic mean	252.67-271.81	238.24-250.02
Median	262.5	236
95% CI for the median	-254.54270.45	228.84-246.00
Standard deviation	72.68	44.73
Relative standard deviation	0.27 (27.72%)	0.18 (18.33%)
Variance ratio	2.63	

CAP, controlled attenuation parameter; CCC, concordance correlation coefficient; UAP, ultrasound attenuation parameter.

^†^Deming regression; *x*: CAP and *y*: UAP.
